# Artificial Intelligence for Optical Coherence Tomography in Glaucoma

**DOI:** 10.1167/tvst.14.1.27

**Published:** 2025-01-24

**Authors:** Mak B. Djulbegovic, Henry Bair, David J. Taylor Gonzalez, Hiroshi Ishikawa, Gadi Wollstein, Joel S. Schuman

**Affiliations:** 1 Glaucoma Service, Wills Eye Hospital, Philadelphia, PA, USA; 2 Department of Ophthalmology, Sidney Kimmel Medical College at Thomas Jefferson University, Philadelphia, PA, USA; 3 Hamilton Eye Institute, University of Tennessee Health and Science Center, Memphis, TN, USA; 4 Oregon Health Science University, Portland, OR, USA; 5 Drexel University School of Biomedical Engineering, Science and Health Studies, Philadelphia, PA, USA

**Keywords:** artificial intelligence (AI), optical coherence tomography angiography (OCTA), optical coherence tomography (OCT), glaucoma

## Abstract

**Purpose:**

The integration of artificial intelligence (AI), particularly deep learning (DL), with optical coherence tomography (OCT) offers significant opportunities in the diagnosis and management of glaucoma. This article explores the application of various DL models in enhancing OCT capabilities and addresses the challenges associated with their clinical implementation.

**Methods:**

A review of articles utilizing DL models was conducted, including convolutional neural networks (CNNs), recurrent neural networks (RNNs), generative adversarial networks (GANs), autoencoders, and large language models (LLMs). Key developments and practical applications of these models in OCT image analysis were emphasized, particularly in the context of enhancing image quality, glaucoma diagnosis, and monitoring progression.

**Results:**

CNNs excel in segmenting retinal layers and detecting glaucomatous damage, whereas RNNs are effective in analyzing sequential OCT scans for disease progression. GANs enhance image quality and data augmentation, and autoencoders facilitate advanced feature extraction. LLMs show promise in integrating textual and visual data for comprehensive diagnostic assessments. Despite these advancements, challenges such as data availability, variability, potential biases, and the need for extensive validation persist.

**Conclusions:**

DL models are reshaping glaucoma management by enhancing OCT's diagnostic capabilities. However, the successful translation into clinical practice requires addressing major challenges related to data variability, biases, fairness, and model validation to ensure accurate and reliable patient care.

**Translational Relevance:**

This review bridges the gap between basic research and clinical care by demonstrating how AI, particularly DL models, can markedly enhance OCT's clinical utility in diagnosis, monitoring, and prediction, moving toward more individualized, personalized, and precise treatment strategies.

## Introduction

Marked advances have been made in recent years in artificial intelligence (AI) technologies, and applications in healthcare. Among the various fields benefiting from these advances, ophthalmology has seen substantial innovations. AI has had a role in imaging-based diagnostics and disease monitoring. The clinical deployment of AI in ophthalmology goes back to 2018 when the US Food and Drug Administration (FDA) approved the use of a computer vision algorithm for the diagnosis of diabetic retinopathy—the first such AI tool approved by the FDA for the diagnosis of any disease.^1^ This landmark approval marked a pivotal moment in AI applications in healthcare. But even before then and certainly since, proposed and tested ophthalmic applications of AI have proliferated.^2^

AI broadly refers to software-based systems capable of performing tasks that typically require human intelligence and behavior, ranging from decision making to object detection through computer vision and language manipulation, often achieving results that surpass human capabilities or detecting nuances that are imperceptible to humans. Within the field of AI, machine learning (ML) refers to algorithmic models that learn from and make predictions or decisions based on data. In contrast, deep learning (DL) is a subset of ML that involves neural networks with multiple layers (hence “deep”) that enable the modeling of complex patterns and high-level abstractions in data. These DL models, particularly well-suited for analyzing unstructured data such as images and videos, are increasingly being applied to enhance optical coherence tomography (OCT) image analysis in ophthalmology and are particularly useful in the management of glaucoma.^3^

Our review aims to explore the application of various DL models, including convolutional neural networks (CNNs), recurrent neural networks (RNNs), generative adversarial networks (GANs), autoencoders, and large language models (LLMs), to enhance the diagnosis, monitoring, and treatment of glaucoma. These models leverage OCT's capacity to provide high-resolution, noninvasive imaging of the retina, enabling precise structural analyses, tracking of disease progression, and identification of novel biomarkers.

By reviewing the distinct strengths of each DL model, the study highlights their complementary roles in addressing critical challenges in glaucoma management, such as image quality limitations, variability in diagnostic criteria, and the need for personalized approaches. For example, CNNs excel at detecting subtle glaucomatous changes in OCT images, whereas RNNs are well-suited for analyzing longitudinal data to understand disease trajectories. GANs improve OCT image quality, extending the utility of legacy imaging systems, and autoencoders streamline feature extraction and dimensionality reduction, enhancing diagnostic precision. Meanwhile, LLMs and foundational models exemplify AI's potential to integrate multimodal data, providing comprehensive insights that reflect clinical decision-making processes.

Our review also considers the challenges associated with implementing AI-driven OCT systems, such as regulatory hurdles, bias in training datasets, and technical limitations in current imaging technologies. In response, emerging solutions, including federated learning, enhanced model validation across diverse populations, and AI-augmented intraoperative OCT, are proposed to address these barriers. By integrating these advanced technologies, the field is moving toward predictive and personalized glaucoma care, paving the way for earlier interventions and improved patient outcomes. This work contributes to the growing body of research aimed at harnessing the potential of AI to transform OCT-based glaucoma management while acknowledging the complexities and limitations inherent in this rapidly evolving domain.

## Review of DL Models Applied to OCT in Glaucoma Management

In 2022, LLMs became more accessible with the dissemination of ChatGPT and similar models, showcasing their transformative potential.^4^^,^^5^ In the following section, we will explore several models, each offering unique methods for processing data and playing vital roles in glaucoma management (see the Table). We will introduce leading methods, such as CNNs and RNNs, known for their powerful data processing capabilities, and GANs and autoencoders, which excel in creating and refining complex datasets. This review focuses on these several prominent DL models as they have shown the most promise and practical application in recent OCT image analysis studies. These models were selected based on a narrative review of the literature, prioritizing studies that demonstrated robust performance in OCT-related tasks, such as image enhancement, segmentation, and disease classification. Whereas the field of DL is rapidly evolving with a wide variety of model architectures and innovations, these selected models represent techniques that are currently popular and have been extensively explored in medical imaging. We will also review recent advancements in AI technology and highlight their emerging contributions to ocular healthcare, such as large language models and foundational models. Their innovative approach to natural language processing opens new possibilities for integrating patient data and clinical insights. As we delve into these models, we will highlight how each contributes to advancing glaucoma management and improving patient outcomes, underscoring the transformative potential of AI in this field.

**Table.** Overview of Deep Learning Models Applied to Optical Coherence Tomography (OCT) in Glaucoma Management

Deep Learning Model	Primary Function	Specific Applications in OCT for Glaucoma	Notable Studies
Convolutional neural networks (CNNs)	Image segmentation and classification	Segmenting retinal layers, detecting glaucomatous damage, classifying eyes	Ran et al., Maetschke et al., Lee et al., Chen et al.
Recurrent neural networks (RNNs)	Temporal analysis	Analyzing sequential OCT scans for disease progression	Ashtari-Majlan et al., Gheisari et al.
Generative adversarial networks (GANs)	Synthetic image generation and data augmentation	Enhancing image quality, increasing database depth, converting TDOCT to SDOCT quality	Lazaridis et al., Thakoor et al.
Autoencoders	Feature extraction and dimensionality reduction	Extracting and compressing features from OCT images to improve disease progression assessment	Shon et al., Bowd et al., Panda et al.
Large language models (LLMs)	Natural language processing	Integrating textual and visual data for comprehensive diagnostic assessment	Delsoz et al., Huang et al., Ghalibafan et al., Antaki et al.

### Convolutional Neural Networks

CNNs are the most common type of DL model used for processing OCT images in glaucoma diagnosis. CNNs are particularly suited for image analysis due to their ability to automatically and adaptively learn spatial hierarchies of features, making them effective for detecting subtle patterns associated with glaucoma.^6^^,^^7^ These models excel in segmenting retinal layers, detecting glaucomatous damage, and differentiating between normal and pathological states.

Different types of OCT images have been utilized to develop DL algorithms for glaucoma diagnosis. These include the OCT conventional report, cross-sections, 3D volumetric scans, anterior segment OCTs, and OCT angiography (OCTA) images.^8^^,^^9^ Each type of image provides unique information, enhancing the CNNs’ ability to detect glaucoma accurately. For instance, DL models trained with images extracted from the OCT single report can achieve high accuracy in detecting glaucoma. A recent review of published CNNs demonstrated high area under the curve (AUC) scores ranging from 0.78 to 0.99, underscoring the effectiveness of these models in differentiating glaucomatous eyes from healthy eyes and predicting retinal nerve fiber layer (RNFL) thickness across different glaucoma stages.^10^ These results highlight CNNs’ potential to significantly enhance glaucoma management by providing precise and early detection capabilities.

Ran et al. introduced a 3D CNN system that utilizes volumetric OCT data to analyze the posterior segment of the eye, demonstrating its potential to accurately identify glaucomatous optic neuropathy by understanding complex retinal structures.^11^ In their study, the 3D DL system achieved an area under the receiver operation characteristics curve (AUROC) of 0.969 (95% confidence interval [CI] = 0.960–0.976), a sensitivity of 89% (95% CI = 83–93), a specificity of 96% (95% CI = 92–99), and an accuracy of 91% (95% CI = 89–93) in the primary validation dataset. Similar performance metrics were observed in external validation datasets, with AUROCs ranging from 0.893 to 0.897 and accuracies between 80% and 86%.^11^ Building on this foundation, their subsequent work expanded the model into a multi-task CNN system capable of detecting both glaucomatous and myopic features with high precision across multiple centers.^12^ In internal validation, the model achieved an AUROC of 0.949 compared to 0.913 for average RNFL thickness (*P* < 0.001) in detecting glaucomatous optic neuropathy. External testing across five independent datasets demonstrated generalizability, with subgroup analysis revealing superior performance in eyes without myopic features (AUROC = 0.965 vs. 0.883, *P* < 0.001) in one dataset. For detecting myopic features, the model showed robust performance, with AUROC values ranging from 0.855 to 0.896 across all datasets.^12^ These advancements underscore the model's progression from a conceptual framework to a robust diagnostic tool, highlighting its ability to detect subtle changes in the optic nerve head and retinal layers, thus enhancing diagnostic capabilities beyond traditional methods.

In another significant development, Maetschke et al. introduced a CNN that classifies eyes using unsegmented OCT volumes.^13^ This approach diverges from traditional methods that require manual segmentation of OCT images, which is time-consuming and subject to human error. Instead, Maetschke et al.’s model processes raw OCT data, allowing for a more streamlined and automated classification process. A notable feature of their approach is class activation maps (CAMs), which provide insights into which regions of the OCT volume are critical for glaucoma detection. The DL model achieved an AUC of 0.94, significantly outperforming the best-performing classical ML method, logistic regression, which achieved an AUC of 0.89.^13^ The CNN identified key anatomic regions, such as the peripapillary RNFL, neuroretinal rim, optic disc cupping, and the lamina cribrosa as significant for glaucoma classification. These areas correspond to established clinical markers, including increased cup volume, cup diameter, and RNFL and neuroretinal rim thinning, particularly in the superior and inferior regions of the optic nerve.

In a groundbreaking advancement, Lee et al. developed a CNN-based DL model that significantly enhances the detection of glaucomatous changes using spectral domain optical coherence tomography (SD-OCT) images.^14^ SD-OCT provides high-resolution, cross-sectional images of the retina, which are crucial for identifying subtle structural changes indicative of glaucoma. Unlike traditional diagnostic methods that rely on isolated structural features, Lee et al.’s model leverages CNNs to process comprehensive SD-OCT data that merged ganglion cell-inner plexiform layer and RNFL OCT maps to distinguish glaucomatous damage more effectively. Their DL system reached an AUC of 0.990 (95% CI = 0.975–1.000) with a sensitivity of 94.7% and a specificity of 100.0%. Ultimately, this model demonstrated superior sensitivity and specificity, effectively identifying early glaucomatous damage that was not accounted for by previous AI systems.

Dr. Stephen M. Drance was a pioneer in establishing the importance of structure-function relationships in glaucoma. Through his clinical research, Drance highlighted that functional vision field loss often corresponds to structural changes in the optic nerve head (ONH) and surrounding regions.^15^ He observed that optic nerve hemorrhages frequently precede progressive visual field deterioration, serving as an early indicator of glaucomatous damage. Drance's studies identified these hemorrhages as predictive markers of future vision loss, as they often appeared before other glaucomatous changes in the retinal nerve fiber layer or ONH topography. His findings laid the groundwork for advancing structure-function mapping techniques that integrate anatomic and functional data, providing a more comprehensive understanding of glaucoma progression. Building on Drance's principles, the seminal work of Garway-Heath and colleagues in 2000 provided a standardized map to align specific visual field test points with corresponding regions of the ONH.^16^ The Garway-Heath map has since been essential in research and clinical practices, enhancing the precision of correlating structural metrics from imaging modalities like 3D OCT with functional outcomes. Following these developments, Chen et al. further advanced the field by developing a DL model based on the spatial relationship of structure and function through mapping the visual field test points to optic ONH regions based on 3D OCT data.^17^ This study achieved accurate structure-function mapping without relying on prior knowledge, segmentation, or assumptions about the relationship, thereby minimizing bias and segmentation errors. By using occlusion analysis, the model identified specific ONH regions that significantly contribute to visual field sensitivity, aligning well with clinical expectations and offering a robust, unbiased approach to understanding glaucoma-related visual field loss.

As OCTA continues to be integrated into clinical practice, the use of AI platforms to analyze OCTA-derived biomarkers is becoming increasingly important.^18^^,^^19^ A recent study by Ninomiya et al. demonstrates this by investigating the relationship between foveal avascular zone (FAZ) parameters, measured through AI-assisted OCTA, and visual field deterioration in patients with open-angle glaucoma. The study shows that a DL system can predict changes in FAZ that are closely linked with the progression of VF defects. Specifically, FAZ parameters, such as area, circularity index, and perimeter, were significantly associated with total deviation (TD) and TD slope in the inferocentral quadrant (β = −0.244 to −0.168, *P* < 0.001), highlighting their relevance to glaucomatous damage.^19^ Their findings suggest that FAZ parameters, such as area, circularity index, and perimeter, could serve as valuable biomarkers for ocular hypoperfusion and glaucomatous damage. For instance, FAZ area enlargement was associated with the female gender (β = 0.242, *P* = 0.003), whereas loss of FAZ circularity was linked to aging (β = −0.188, *P* < 0.001) and sleep apnea syndrome (β = −0.261, *P* = 0.031).^19^ The study further highlights associations between FAZ characteristics and systemic factors, such as aging, female gender, and sleep apnea syndrome, suggesting that there is a multifactorial nature of glaucoma progression. This research illustrates the potential of AI-enhanced OCTA in advancing our understanding and monitoring of glaucomatous changes, positioning FAZ parameters as promising indicators for early detection of open-angle glaucoma.

CNNs are increasingly applied to anterior segment optical coherence tomography (AS-OCT) images, filling a crucial diagnostic gap in glaucoma care by addressing the limitations of gonioscopy—a critical but often underutilized component of the glaucoma examination. CNN-based algorithms have been developed to analyze AS-OCT images, enabling the automatic detection of scleral spur, gonioscopic angle closure, and peripheral anterior synechiae, which are essential for assessing glaucoma risk.^20^^,^^21^ For example, a study conducted in China and Singapore developed three CNN classifiers to differentiate among control, primary angle-closure suspect (PACS), primary angle closure (PAC), and primary angle-closure glaucoma (PACG).^22^ The study trained and validated the models using patient data from China and tested the generalizability on an external dataset consisting of patients from Singapore. Classifier 1 distinguished control from PACS and PAC/PACG, achieving an AUC of 0.96 on a regional validation set but dropping to 0.84 on an external test set. Classifier 2, which separated control from primary angle-closure disease (PACD), demonstrated the most robust performance, with AUCs of 0.96 and 0.95 on validation and external test sets, respectively. Classifier 3, aimed at differentiating PACS from PAC/PACG, showed the weakest performance, with AUCs of 0.83 and 0.64 on validation and test sets.^22^ In another study involving a multicenter international effort to develop a deep learning classifier for detecting gonioscopic angle closure, the model demonstrated consistent performance across diverse populations, achieving areas under the AUCs of 0.917 in the Chinese American Eye Study, 0.894 in a predominantly Chinese Singaporean cohort, and 0.922 in a multiethnic cohort at the University of Southern California.^23^ These advancements potentially improve the accessibility and consistency of angle assessment by reducing the dependency on examiner expertise and the subjective nature of gonioscopy. Notably, FDA-approved devices, such as ANTERION (Heidelberg Engineering, Heidelberg, Germany), now integrate AI algorithms, streamlining the analysis process and allowing for more efficient and convenient diagnostic workflows.

### Recurrent Neural Networks 

RNNs are a class of artificial neural networks designed to recognize patterns in data sequences. Unlike traditional feedforward neural networks such as CNNs, RNNs have connections that form directed cycles, allowing them to maintain a memory of previous inputs. This architecture makes RNNs particularly effective for tasks involving temporal or sequential data, such as medical imaging for disease progression analysis. In the context of OCT for glaucoma management, RNNs can analyze a sequence of OCT scans over time to track changes in the ONH and RNFL, which are critical for monitoring the progression of glaucoma. This capability allows for a more dynamic and nuanced understanding of the disease's natural history.

For instance, Ashtari-Majlan et al. used a spatial-aware transformer framework, a sophisticated hybrid of RNN and transformer models, to enhance glaucoma diagnosis using 3D OCT images.^24^ This approach capitalized on the ability of RNNs to process temporal sequences, effectively capturing the progression of glaucoma by analyzing changes over time in the structural data provided by OCT scans. The framework demonstrated remarkable performance, achieving an F1 score of 93.58%, a Matthews Correlation Coefficient of 73.54%, and an AUC of 95.24%.^24^ Their study demonstrated that incorporating temporal dynamics into the analysis improved diagnostic accuracy and a more comprehensive understanding of glaucoma progression than static image analysis. Additionally, a study by Gheisari et al. combined CNNs with RNNs to enhance glaucoma detection using OCT images.^25^ This hybrid approach effectively integrated CNNs to extract spatial features with RNNs to capture temporal dependencies, achieving an average F measure of 96.2% in separating glaucoma from healthy eyes, compared to 79.2% achieved by the standalone CNN model.^25^ By incorporating temporal dependencies often associated with disease progression, this method underscores the utility of dynamic analysis in advancing glaucoma diagnostics. However, despite the advantages offered by RNNs, the absence of a standardized definition for glaucoma progression introduces variability that can complicate the comparison of results across studies. Furthermore, simpler statistical approaches, such as trend-based Guided Progression Analysis, remain valuable in clinical settings, providing practical and interpretable insights.^26^^,^^27^ These methods may complement more complex models, offering a balanced approach to tracking disease progression and enhancing their applicability in diverse clinical contexts.

### Generative Adversarial Networks 

GANs continue to significantly enhance the field of OCT in glaucoma management, offering innovative solutions for improving image quality and data reliability. GANs augment and synthesize OCT images to increase the depth of images used in training DL models, enhancing the robustness of AI algorithms crucial for accurate diagnosis and progression tracking of glaucoma.^28^ This is especially useful in trials reliant on time-domain OCT (TD-OCT), where GANs help transform these images to the quality of SD-OCT, thus increasing statistical power and reducing the need for large cohorts.^29^ Historically, clinical trials like the UK Glaucoma Treatment Study have relied on TD-OCT, which suffers from lower signal-to-noise ratios and image quality, limiting the statistical power to detect meaningful treatment effects. A study by Lazaridis et al. leveraged a novel GAN-based approach to perform a quality transfer from SD-OCT to TD-OCT, significantly enhancing TD-OCT image fidelity.^29^ By integrating multiple GAN outputs through label fusion and image stitching, the authors achieve RNFL segmentations that approach the anatomic accuracy of SD-OCT, allowing for more reliable measurement and analysis. This advancement in image quality facilitates more effective separation of treatment and placebo groups in clinical trials and makes high-resolution OCT data more accessible in settings where SD-OCT is unavailable. Ultimately, the study demonstrates that GANs can empower TD-OCT to reach near-SD-OCT quality, boosting the statistical robustness of clinical trials and improving diagnostic applications in glaucoma research.^29^

Generative models like GANs also address the challenge of collecting large clinical datasets for training DL networks by synthesizing OCT images. A recent study demonstrated that DL networks trained with synthetic circumpapillary ONH OCT images achieved diagnostic performance comparable to those trained on real images.^30^ Notably, the best-performing network trained on synthetic data achieved an AUC of 0.97 (95% CI = 0.95–0.99) on an internal test set and 0.90 (95% CI = 0.87–0.93) on an external set, comparable to AUCs of 0.96 (95% CI = 0.94–0.99) and 0.84 (95% CI = 0.80–0.87) for networks trained with real images.^30^ CAMs further showed that synthetic images contributed to glaucoma detection in a manner consistent with real images. Furthermore, GANs are leveraged to enhance portable OCT device images, making them more accessible in low-resource settings and thereby broadening the scope for early detection and management of glaucoma worldwide.^31^ The ability to generate synthetic images also aids in training DL models for specific tasks like detecting angle closure in glaucoma, as demonstrated by the generation of anterior segment OCT images for training purposes.^32^ By using GANs, researchers and clinicians can create rich, high-quality datasets that could improve diagnostic algorithms and clinical outcomes.

### Autoencoders

Autoencoders are another type of DL that can enhance the analysis of OCT images through advanced dimensionality reduction and feature extraction. These neural network architectures compress complex OCT data into a lower-dimensional space, isolating critical features that improve diagnostic accuracy and disease progression assessment. In a study by Shon et al., variational autoencoders were used to analyze anterior segment OCT images, uncovering hidden patterns and features that enhance the ability to differentiate between various stages of glaucoma and improve diagnostic precision.^33^ The model analyzed data from 2111 eyes, successfully identifying latent variables that captured features such as anterior chamber area, corneal curvature, and pupil size. One of these variables, representing complex interactions among multiple anterior segment structures, was significantly smaller in PACG eyes compared with PAC eyes (*P* = 0.015), highlighting its potential for detecting subtle differences in disease progression. Similarly, Bowd et al. utilized an autoencoder to extract and compress features from OCT images to enhance the classification and improve the assessment of glaucomatous changes.^34^ The study demonstrated that the DL-based regions of interest (ROIs) identified progression in glaucoma eyes with a sensitivity of 90%, significantly higher than the 63% sensitivity achieved using global RNFL thickness measurements. Additionally, the ROIs showed faster rates of structural change in progressing eyes compared to conventional measurements (−1.28 µm/year vs. −0.83 µm/year), emphasizing the method's potential for earlier and more precise detection of disease progression.^34^ Furthermore, Panda et al. demonstrated the use of autoencoder networks in SD-OCT image analysis, effectively reducing dimensionality and enhancing image quality for improved disease detection and characterization of novel biomarkers for glaucoma.^35^ The study achieved a diagnostic accuracy of 92% with a sensitivity of 90% at 95% specificity, demonstrating the network's robust performance. By altering principal components in the latent space, the researchers illustrated how ONH morphology shifts from a “nonglaucoma” to a “glaucoma” condition, linking these structural changes to clinical observations and identifying novel biomarkers for glaucoma diagnosis.^35^ By concentrating on the most relevant features, these studies illustrate how autoencoders can improve diagnostic precision. Additionally, autoencoders can reduce the required training set size and computing resources, making OCT analysis more accessible and efficient. Integrating autoencoders with other DL models could offer a comprehensive approach to OCT image analysis, providing detailed insights into the structural characteristics associated with glaucoma and other ophthalmic conditions.

### Large Language Models 

LLMs represent a remarkable advancement in DL, fundamentally altering how machines understand and generate human language. Their emergence can be traced back to the groundbreaking work by researchers at Google AI, who introduced the transformer architecture in their seminal paper “Attention Is All You Need.”^36^ This innovative architecture revolutionized natural language processing by relying entirely on attention mechanisms, eliminating the need for recurrent and CNNs.^36^ Transformers enable LLMs to process vast amounts of text data and learn complex language patterns and contextual nuances with impressive accuracy and coherence. As a result, LLMs have revolutionized industries and empowered professionals and laypersons to interact with technology more intuitively and innovatively.^37^ The potential of LLMs to transform industries is particularly notable in fields such as ophthalmology, where they offer unique opportunities to revolutionize digital eye care, address clinical workflow inefficiencies, and enhance patient experiences.^38^

### Revolutionizing Accessibility With ChatGPT

The release of ChatGPT by OpenAI marked a significant milestone in making LLMs widely accessible, revolutionizing the AI industry. Launched in November 2022, ChatGPT brought LLM capabilities directly to the public, allowing users from various fields to interact with AI conversationally. This user-friendly interface and the model's ability to understand and generate human-like text democratized access to advanced AI, enabling individuals and businesses to leverage its capabilities without needing specialized technical expertise.

GPT-4 was released on March 14, 2023, as the next iteration of OpenAI's large language model, featuring improvements in capabilities and performance compared to its predecessor, GPT-3.5, which popularized ChatGPT.^4^ The main advancement in GPT-4, aside from enhanced accuracy and reasoning, was its distinction as the first mainstream multimodal large language model. GPT-4 can process and generate text while interpreting images, allowing it to understand and respond to visual inputs alongside text.^4^ Many studies have assessed GPT-4’s capabilities in various fields, with “The Dawn of the LLMs” paper being the first comprehensive study to evaluate GPT-4’s vision capabilities in various settings, including its ability to process medical images.^39^

### Application of Language Models for OCT in Glaucoma Management

The potential of LLMs in healthcare has been significantly highlighted by their application in ophthalmology, particularly in diagnosing glaucoma. Whereas transformative, applying these DL models to interpret OCT scans for glaucoma management has not been explored despite their applications in analyzing OCT images for other retinal diseases, such as macular degeneration. This represents a significant opportunity for future research. In this section, we highlight examples of how LLMs and other language models are applied in ophthalmology with OCT as the primary data input, demonstrating innovations that may be transferable to glaucoma diagnosis and management.

A recent study evaluated the effectiveness of GPT-4 in diagnosing vitreoretinal diseases using textual health information and OCT images.^40^ Despite the model's ability to diagnose common conditions, such as posterior vitreous detachment, non-exudative age-related macular degeneration, and retinal detachment, it was inaccurate, and its effectiveness was limited in more complex, open-ended medical scenarios.^40^ Ultimately, the study highlights the potential of LLMs in processing OCT images with other patient information within ophthalmology.

Another study published in *JAMA Ophthalmology* evaluated another type of language model, the Gemini Pro vision-language model (VLM), for its ability to detect features of macular diseases using OCT scans.^41^ The Gemini Pro VLM was tasked with identifying ten key pathological features, providing diagnoses, making referral recommendations, and suggesting treatments. Despite its innovative design, the VLM achieved a mean F1 score of only 10.7% in feature detection, indicating significant limitations in its vision capabilities.^41^ Correct diagnoses were achieved in 34% of cases, whereas referral recommendations were accurate in 56% of instances.^41^ Notably, the model demonstrated high internal concordance, correctly aligning referral and treatment recommendations in 96% and 98% of cases, respectively.^41^ This suggests that whereas the Gemini Pro VLM excels in language processing and logical consistency, it struggles with the visual complexity of OCT scans.

### Future Directions

As we look ahead, future directions of AI for OCT in glaucoma management are emerging, (see the Fig.). Integrating AI and DL models with OCT is poised to revolutionize glaucoma management by advancing beyond diagnostic capabilities to include predictive analytics and personalized medicine. By leveraging the vast amounts of data generated by OCT and combining it with sophisticated AI algorithms, clinicians can move toward predicting disease progression, customizing treatment plans, and improving patient outcomes. This shift toward predictive and personalized approaches is expected to transform how glaucoma is managed, providing earlier interventions and tailored therapies that cater to individual patient needs. Moreover, the efficiency gains from AI integration may streamline clinical workflows, allowing clinicians to process and interpret OCT data more quickly and accurately. This efficiency is crucial in busy clinical settings, where time and accuracy are paramount.

**Figure.**
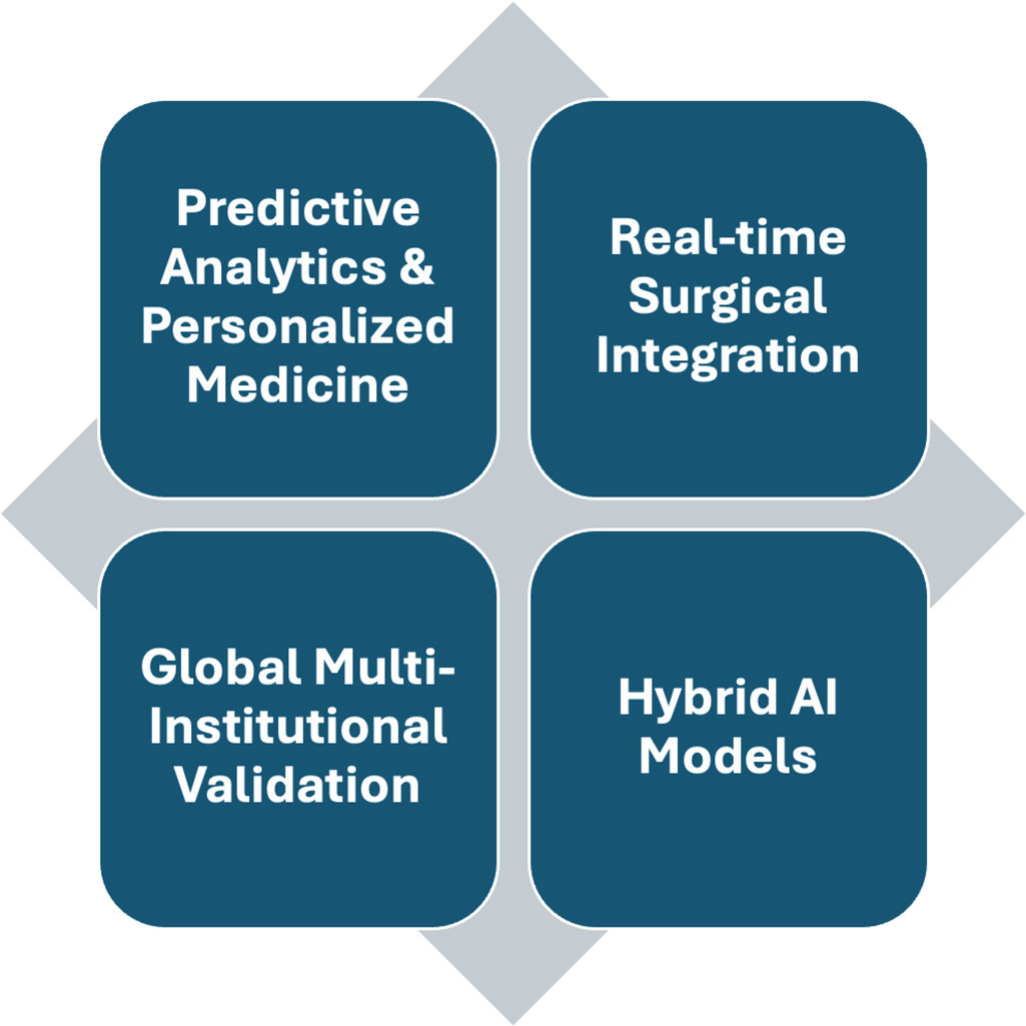
Future directions of AI for OCT in glaucoma management.

Foundation models are large-scale, pretrained neural networks that serve as versatile bases for various downstream tasks. By leveraging extensive datasets and self-supervised learning techniques, these models capture a wide range of features, enabling efficient adaptation to specific applications with minimal additional training. In ophthalmology, RetFound exemplifies this approach, having been trained on 1.6 million unlabeled retinal images to develop a comprehensive understanding of retinal structures and pathologies.^42^ RetFound's adaptability is particularly beneficial for glaucoma detection. A study demonstrated that fine-tuning RetFound with a relatively small dataset of 2000 OCT RNFL scans over 50 training epochs achieved an AUC of 0.91, indicating high accuracy in distinguishing glaucomatous from non-glaucomatous eyes.^43^ Building on this concept, EyeFound advances foundation models in ophthalmology by enabling multimodal learning across diverse imaging modalities. Trained on 2.78 million images from 227 hospitals covering 11 ophthalmic imaging techniques, EyeFound facilitates generalizable representations for tasks ranging from disease diagnosis to visual question answering, even excelling in detecting rare diseases.^44^ EyeFound has outperformed RetFound in diagnosing eye diseases and predicting systemic conditions, showcasing its potential to broaden AI's clinical utility in ophthalmology. The impact of RetFound and other foundational models is notable as it requires fewer labeled samples for fine-tuning, leading to accelerated algorithm development.

Recent advancements have demonstrated the successful integration of AI with OCT, particularly in intraoperative settings. One notable implementation is the incorporation of AI-driven OCT into surgical microscopes, enabling real-time image analysis and guidance during surgery.^45^^–^^47^ Intraoperative optical coherence tomography (iOCT) exemplifies 4D imaging by providing real-time, high-resolution visualization during ophthalmic surgeries. For instance, in anterior segment procedures like Descemet Stripping Automated Endothelial Keratoplasty (DSAEK), iOCT allows surgeons to confirm proper graft positioning and adherence, potentially reducing the risk of graft detachment.^45^ In pediatric glaucoma surgery, it aids in precise needle placement during ab-interno trabeculotomy and helps visualize Schlemm's canal.^46^ This technology enhances surgical decision making and improves patient outcomes by offering immediate feedback on tissue and instrument positioning, with studies showing it impacts surgical decisions in 29% of posterior segment cases and 43% of anterior segment cases.^47^ As it requires 4D imaging, where the fourth dimension is time, this capability allows for enhanced intraoperative imaging, significantly aiding precision and safety in surgeries. The incorporation of AI holds the potential to further elevate these capabilities. For instance, CNNs can drastically enhance image processing through real-time analysis and precise pattern recognition, enabling surgeons to detect minute details and variations in tissue structure. RNNs and autoencoders could advance predictive analytics and anomaly detection by identifying subtle changes in imaging data over time, allowing for proactive adjustments during surgery. GANs offer groundbreaking potential to simulate and refine surgical strategies by generating realistic imaging data, thereby improving the training and robustness of AI models for diverse surgical applications. Last, LLMs can facilitate advanced data interpretation and decision-making processes, ensuring comprehensive real-time guidance and insights for surgical teams. For instance, in the not-so-distant future, surgeons could interact directly with the LLM using natural language and spoken voice during procedures to assist in decision making, benefiting from the model's extensive knowledge and analysis capabilities. Furthermore, developing hybrid models that combine the strengths of each type of DL model is crucial for maximizing the potential of AI-driven OCT systems. Collectively, these AI technologies promise to revolutionize the use of OCT in surgical settings, leading to more informed and precise interventions.

The continued development of DL architectures is essential to maximizing the potential of AI in OCT and glaucoma management. This includes the iterative refinement of existing models to enhance their performance in clinical applications. Internal validation within research groups is an essential first step in this process, ensuring that models meet initial standards of accuracy and reliability. However, to truly leverage the power of AI, there must be a concerted global and multi-institutional effort to externally validate these models across diverse datasets and clinical environments. This approach would enhance the robustness of AI-driven solutions and facilitate their adoption in different healthcare settings. Open collaboration and data sharing between institutions are likely key to achieving these goals, as they enable researchers to build on collective insights and refine algorithms more effectively. By fostering such collaborative efforts, the healthcare community can accelerate the development of AI technologies that are both broadly applicable and precisely tailored to meet the specific needs of glaucoma management.

As the capabilities of AI in image analysis continue to evolve, the integration of Federated Learning (FL) into OCT-based glaucoma management systems offers a critical pathway to overcoming data privacy challenges while enhancing the robustness and generalizability of AI models. FL enables the creation of large-scale, diverse datasets by allowing multiple institutions to collaborate without sharing raw data.^48^^,^^49^ By facilitating secure data sharing, FL accelerates research and ensures that the resulting AI-driven tools are adaptable to diverse clinical environments. Integrating FL with continuous learning fosters a dynamic ecosystem where AI models are perpetually updated and refined with new data, ensuring they remain adaptive and relevant in real-time applications. This decentralized approach may be key for harnessing the full potential of AI in healthcare, allowing for more accurate diagnostics, personalized treatments, and improved patient outcomes in glaucoma care. As we look ahead, the synergy between advanced AI techniques and privacy-preserving frameworks like FL will be instrumental in shaping the future of glaucoma management, ensuring that innovations in OCT and AI are cutting-edge and data-secure.

### Challenges and Considerations in Implementing AI for OCT

Despite the transformative potential of AI and DL models in healthcare, several challenges remain unresolved, particularly concerning the regulatory approval and deployment of AI algorithms. Three prominent issues include (1) the lack of a consensual definition of glaucoma, which complicates the standardization of diagnostic criteria; (2) the unreliability and bias of human graders when defining ground truth validation datasets, which undermines the accuracy and generalizability of AI models; and (3) the sparsity of publicly available real-world data that encompasses diverse population characteristics and adheres to established data quality standards.^50^^,^^51^ These challenges are further highlighted by the limited size and representativeness of datasets used to train AI models, often leading to overfitting and reduced generalizability across different populations and clinical settings.^51^ The presence of bias, whether from data annotation or inherent in the training datasets, can significantly skew AI model predictions, impacting their fairness, reliability, and effectiveness in clinical practice.

Not with standing the advantages of OCT in glaucoma management, there are certain considerations regarding the current use of imaging technology. Clinician expertise and experience are needed to interpret OCT images, which can lead to variability in assessments. Factors such as pupil size, dry eye, cataracts, anatomic variations, advanced ophthalmic pathology, and ambiguity of retinal layer boundaries due to pathologies such as edema can impact OCT measurements.^52^ These factors induce algorithmic segmentation errors, which are inaccuracies in how OCT software algorithms delineate the various anatomic layers of the retina or other structures within the eye. The factors above impede the ability of OCT to recognize differences in tissue reflectivity to visualize anatomic layers. Technical issues such as motion artifacts and scan placement mistakes can also impact OCT results.^53^ Different types of artifacts, such as movement, flashing, and device restrictions, may be present and affect result interpretation.^54^ Low-resource settings and crowded clinics are common places for suboptimal scan positioning and misalignment, which can lead to poor quality pictures.

These issues can potentially be addressed by the most recent AI models and developments in both OCT hardware and software. AI-driven improvements in OCT technology include better image resolution and faster scanning speeds that minimize artifacts in the raw data. As OCTs have become commonplace in facilities internationally, it has become possible to collect and share large-scale datasets of these images. By analyzing massive databases of OCT images and clinical vignettes, AI models can correct errors in real-time and improve the segmentation process, reduce artifacts, and adjust for patient movements. Together, these advancements can perform more detailed analyses of the eye's anatomy, offering insights previously challenging to obtain or invisible to the human clinician.^55^ Importantly, AI is capable of more than merely enhancing the clarity and quality of OCT output and subsequent analyses. By integrating multimodal clinical data, AI has the potential to simulate the clinician's comprehensive approach, combining data sources to assess disease trajectory and treatment efficacy in a way that mirrors clinical decision making. For instance, it becomes possible to incorporate information from fundus photography, visual field testing, and OCT to produce more comprehensive and precise diagnosis, prognosis, and disease monitoring results. Personalized treatment strategies will become more readily available. In the future, as AI technologies continue to be refined and processing power increases, their applications in ophthalmology will only become more apparent.

## Conclusions

Integrating DL models with OCT represents a transformative advancement in the management of glaucoma. These technologies enhance our diagnostic capabilities and pave the way for predictive analytics and personalized treatment strategies that are more precise and effective. Although the promise of AI in revolutionizing glaucoma care is clear, we must also remain cognizant of the challenges and considerations that come with its implementation. Issues such as data variability, algorithmic biases, and the need for robust validation highlight the complexity of deploying these technologies in a clinical setting. Nevertheless, as we continue to refine these models and address these challenges, the potential of AI and DL to significantly improve outcomes for patients with glaucoma is both profound and inspiring.
